# Supplementary material for: Sports safety matting diminishes cardiopulmonary resuscitation quality and increases rescuer perceived exertion
Source: PLoS One. 2021 Jul 22;16(7):e0254800. doi: 10.1371/journal.pone.0254800 (PMC8297772; doi:10.1371/journal.pone.0254800)
Supplement: S1 File — Additional results relating to magnitude of compression decay and chest leaning present upon each surface. (DOCX) [file pone.0254800.s001.docx]

**S1 File**

As an external measure of fatigue, data pertaining to the effects of each matting surface upon chest compression quality was attained during each compression bout.

**METHODS**

Chest leaning was calculated based as the average minimum depth attained at the end of the release phase of each compression during the 2-min period. Compression decay over time was calculated from the raw data recorded by the mannequin as the mean depth (mm) and rate (min^-1^) of the initial 60 compressions relative to the final 60 compressions within each bout of compressions. Measures of chest leaning were analysed using a One-Way Repeated-Measures Analysis of Variance (ANOVA), with compression decay analysed using a Two-Way ANOVA.

**RESULTS**

*Chest leaning*

Chest leaning (>1 mm) was apparent on all surfaces, but without a significant main effect (F[4, 104]= 2.33, *p*=0.61; η^2^_p_: 0.08; Supplementary Table 1).

*Compression decay*

For the mean depth of the initial *vs.* final 60 compressions, there was a significant main effect for time (F[1, 26] = 68.19, *p*<0.001; η^2^_p_: 0.72) and for surface (F[2.84, 73.77] = 5.99, *p*=0.001; η^2^_p_: 0.19; *post-hoc* tests see: Supplementary Table 1). There was no significant surface × time interaction (F[4, 104]= 1.60, *p*=0.180; η^2^_p_: 0.06). The compression rate for the initial *vs.* final 60 compressions showed a significant main effect for surface (F[4, 104] = 11.19, *p*<0.001; η^2^_p_: 0.30; Supplementary Table 1), but not time (F[4, 104] = 11.19, *p*=0.890; η^2^_p_: 0.00), nor was there a significant interaction between the two (F[4, 104] = 0.404, *p*=0.805; η^2^_p_: 0.02).

**Supplementary Table 1.** Degree of chest leaning among surfaces & compression depth of initial *vs.* final 60 compressions.

|  |  |  | **^(a)^Floor** | | |  | **^(b)^LC** | | |  | **^(c)^LCBB** | | |  | **^(d)^HC** | | |  | **^(e)^HCBB** | | |
| --- | --- | --- | --- | --- | --- | --- | --- | --- | --- | --- | --- | --- | --- | --- | --- | --- | --- | --- | --- | --- | --- |
| **Leaning** |  |  | **3.5** | **±** | **2.3** |  | **3.3** | **±** | **2.8** |  | **2.8** | **±** | **2.0** |  | **3.6** | **±** | **2.9** |  | **4.0** | **±** | **2.7** |
| *CI (95%)* |  |  | 2.6 | - | 4.4 |  | 2.2 | - | 4.4 |  | 2.0 | - | 3.6 |  | 2.5 | - | 4.8 |  | 2.9 | - | 5.0 |
|  |  |  |  |  |  |  |  |  |  |  |  |  |  |  |  |  |  |  |  |  |  |
| **Start (mm)** |  |  | **52.2** | **±** | **3.9** |  | **52.8** | **±** | **4.5** |  | **52.4** | **±** | **3.9** |  | **48.9** | **±** | **4.5** |  | **50.9** | **±** | **4.1** |
| *CI (95%)* |  |  | 50.6 | - | 53.8 |  | 51.1 | - | 54.6 |  | 50.9 | - | 54.0 |  | 47.1 | - | 50.6 |  | 49.3 | - | 52.5 |
|  |  |  |  |  |  |  |  |  |  |  |  |  |  |  |  |  |  |  |  |  |  |
| **End (mm)** |  |  | **47.8** | **±** | **5.1^d^** |  | **47.7** | **±** | **5.9^d^** |  | **47.7** | **±** | **6.4^d^** |  | **45.2** | **±** | **5.5^a,b,c^** |  | **46.4** | **±** | **5.6** |
| *CI (95%)* |  |  | 45.8 | - | 49.8 |  | 45.4 | - | 50.0 |  | 45.2 | - | 50.2 |  | 43.0 | - | 47.4 |  | 44.2 | - | 48.6 |
|  |  |  |  |  |  |  |  |  |  |  |  |  |  |  |  |  |  |  |  | | |
| *Sig.* |  |  | *p <0.001* | | |  | *p <0.001* | | |  | *p <0.001* | | |  | *p <0.001* | | |  | *p <0.001* | | |
|  |  |  |  |  |  |  |  |  |  |  |  |  |  |  |  |  |  |  |  |  |  |

Data are mean ± *SD* (n=27). Floor = concrete floor; LC = low-compliance foam; LCBB = low-compliance foam with backboard; HC = high-compliance foam; HCBB = high compliance foam with backboard. Leaning = mean depth of chest leaning during 2-min bout. Start = mean compression depth during initial 60 compressions; End = mean compression depth during final 60 compressions. *Sig.* = significance level start- versus end-compressions. CI (95%) = 95% confidence interval. ^a^Significantly different versus Floor; ^b^Significantly different versus LC; ^c^Significantly different versus LCBB; ^d^Significantly different versus HC; ^e^Significantly different versus HCBB. Alpha level = <0.05.
