# Supplementary material for: Sports safety matting diminishes cardiopulmonary resuscitation quality and increases rescuer perceived exertion
Source: PLoS One. 2021 Jul 22;16(7):e0254800. doi: 10.1371/journal.pone.0254800 (PMC8297772; doi:10.1371/journal.pone.0254800)
Supplement: S1 Table — Participant descriptive data for all included and excluded participants. (DOCX) [file pone.0254800.s002.docx]

**S1 Table: Individual participant descriptives**

|  | **Gender**  **(m/f)** | **Mass**  **(kg)** | **Height**  **(m)** |
| --- | --- | --- | --- |
|  |  |  |  |
| *Included* |  |  |  |
| **1** | m | 69.1 | 1.75 |
| **2** | f | 68.5 | 1.66 |
| **3** | m | 99.7 | 1.77 |
| **4** | m | 74.4 | 1.82 |
| **5** | f | 100.7 | 1.89 |
| **6** | f | 73 | 1.78 |
| **7** | m | 93.9 | 1.86 |
| **8** | f | 69.1 | 1.72 |
| **9** | m | 69 | 1.84 |
| **10** | m | 91.9 | 1.95 |
| **11** | m | 101.8 | 1.87 |
| **12** | m | 80.3 | 1.78 |
| **13** | m | 85.2 | 1.83 |
| **14** | f | 89.5 | 1.84 |
| **15** | f | 81 | 1.63 |
| **16** | f | 53.8 | 1.66 |
| **17** | m | 91.2 | 1.67 |
| **18** | m | 79.1 | 1.78 |
| **19** | m | 90.5 | 1.76 |
| **20** | f | 77 | 1.83 |
| **21** | m | 79.6 | 1.87 |
| **22** | f | 76.7 | 1.83 |
| **23** | f | 69.4 | 1.70 |
| **24** | f | 64 | 1.62 |
| **25** | f | 65.5 | 1.62 |
| **26** | f | 65.3 | 1.66 |
| **27** | f | 74.3 | 1.69 |
|  |  |  |  |
| **Mean** |  | **79.0** | **1.77** |
| **SD** |  | **12.5** | **0.09** |
|  |  |  |  |
| *Excluded* |  |  |  |
| **28** | f | 61.8 | 1.64 |
| **29** | f | 65.2 | 1.64 |
| **30** | f | 54.8 | 1.71 |
| **31** | f | 44.7 | 1.64 |
| **32** | f | 63.7 | 1.61 |
|  |  |  |  |
| **Mean** |  | **58.0** | **1.65** |
| **SD** |  | **8.5** | **0.04** |
|  |  |  |  |
| *Whole group* |  |  |  |
| **Mean** |  | **75.7** | **1.75** |
| **SD** |  | **14.1** | **0.10** |
|  |  |  |  |
