# Supplementary material for: Sports safety matting diminishes cardiopulmonary resuscitation quality and increases rescuer perceived exertion
Source: PLoS One. 2021 Jul 22;16(7):e0254800. doi: 10.1371/journal.pone.0254800 (PMC8297772; doi:10.1371/journal.pone.0254800)
Supplement: Attachment — Submitted filename: Authors responses to review comments CPR PLoS1.docx [file pone.0254800.s004.docx]

**Sports safety matting diminishes cardiopulmonary resuscitation quality and increases rescuer perceived exertion**

Thomas Kingston, Nicholas B. Tiller, Elle Partington, Mukhtar Ahmed, Gareth Jones, Mark I. Johnson, Nigel A. Callender.

We would like to thank the reviewers for providing comprehensive and informative feedback on the manuscript. Please find below their specific comments along with our annotated responses. We have tracked any revisions to the manuscript by including the relevant line numbers and highlighting changes in **blue** for ease of reference. Note some changes have been made to formatting at the request of the journal.

**Reviewer #1**

Multiple objectives have been reported in the study, however the abstract doesn’t mention about CPR preparedness

*Thank you for highlighting the above. In line with your 3^rd^ comment and those of Reviewer 2, we have removed the section relating to the survey of climbing gyms and CPR preparedness from the manuscript to allow for a more coherent paper. We anticipate publishing this as an independent report.*

There is no limitation mentioned in the manuscript

*Thank you for highlighting, sorry if this was not clear. We ventured some limitations in the ‘Technical Considerations’ section. We have now expanded this slightly and renamed it ‘Limitations’.*

*See line: 298-315.*

Study has multiple objectives and includes a combination of research and survey. I would recommend reporting survey as separate manuscript and keeping objective simple

The survey portion of the study is definitely worth reporting but may be as research letter rather than original investigation.

*Thank you for this recommendation. In order to address this, as you and Reviewer 2 have suggested, we have removed the survey portion of our study and are considering its submission independently. The manuscript has been appropriately revised without the detail relevant to the survey and the relevant tables and figure removed.*

**Reviewer #2**

Overall challenge in the manuscript is it seems there are two main questions/themes the authors are trying to answer , which might confuse the flow and objective of the manuscript.
a. First one is the matting (fold 1 and 2) in the introduction section.
b. Level of CPR preparedness among commercial indoor climbing gymnasiums.

this caused a little bit of a confusion in the sample as it seems both samples are not the same…. In which it is almost having two studies. Have the authors considered having this manuscript focused on only one part, possibly aim 1 and 2? And have #3 separate paper (although may require more sample)?

*Thank you for highlighting the apparent incongruity of the two primary questions and subsequent separate designs and participant groups. As with Reviewer 1, we have removed the CPR preparedness analysis from the manuscript and focussed the current paper just on the matting data. We anticipate publishing this as an independent report.*

The authors used the term Advanced Life Support (ALS), is this only CPR course (Basic Life Support by American Heart Association)? Or Advanced Cardiac Life Support). ? is ALS by ERC similar to BLS in AHA or ACLS?. If it was ALS=ACLS, what was the rationale behind selecting ACLS and not BLS/CPR ?

a. Not to mention that in the second study (survey on preparedness), the authors used the term BLS training instead of ALS. using one consistent term would be better.

*Thanks for highlighting the lack of clarity. Advanced Life Support (ALS) is the European (ERC) equivalent of the AHA Advanced Cardiac Life Support (ACLS) course and is only available to qualified medical professionals (e.g. paramedics, nurses, doctors), and is the recommended qualification for those who may find themselves leading a cardiac arrest team. With ILCOR guidelines shared between the two continents, the course content and level of qualification between ALS and ACLS is essentially identical.*

*ALS qualified participants were used within the experimental portion of our study (examining the influence of matting on chest compression quality) as it may be expected that professional providers may have a greater degree of proficiency and experience in CPR than lay-people. This level of experience was important to us as it may be expected that a greater level of consistency of CPR performance might be provided by this group, limiting the amount of external influences beyond matting type during CPR. We have expanded on this further in our responses to Reviewer 3.*

*Basic life support (BLS) is the basic CPR course open to lay-people (identical to BLS in the United States), covering only chest compressions, simple airway manoeuvres, mouth-mouth ventilation and use of an automated defibrillator. The two levels of qualification (BLS and ALS) do however describe vastly different levels of training (and usually experience) level among those holding the qualification, hence the differentiation. In the original manuscript, we looked at CPR preparedness in gymnasium environments and most of those individuals are trained only in BLS (they are not expected to be trained to an advanced level). However, reference to this analysis has been removed from the manuscript.*

Have the authors thought about using indoor venue staff as subjects instead of recruiting medical personnel , such as critical care, EM, etc? what

*Thank you for highlighting this. We initially attempted to recruit venue staff in addition to our medically-trained participants in order to run a separate, lay-person delivered arm within the study. Unfortunately, our data collection was completed for the medically-trained participants in the final weeks before venues closed due to the Covid-19 Pandemic. We managed to collect data from a very small number of lay-participants (n=4) and since then no further testing has been possible due to: 1) a national lockdown within the UK; 2) constraints within the local venues in relation to the above; and 3) institutional ethics approval which is impossible to attain at present. This is, however, an avenue for a future study, alongside some other key questions the current paper has raised.*

*Although investigating the performance of sports centre staff is highly relevant since they are likely to provide the initial response in the case of a collapsed individual, for the purposes of this study, we wished to control for as many influencing factors as possible in order to elucidate the effect of the supporting surface itself upon chest compression quality. Therefore, by using ALS trained participants we aimed to achieve a high consistency of compression quality within our group than might be delivered by less experienced providers. Our decision making around our participant choices is expanded upon below.*

The study design is a crossover design, meaning subjects did multiple rounds of compressions on various surfaces , wouldn’t that cause some sort of fatigue? Even though they got some rest?

*We agree that multiple rounds of compressions (5 bouts in total) may case some carry-over effects within our participants. However, we presented each surface to participants in a random order, which would mitigate order-related effects, relative to a design where all surfaces were encountered in a fixed sequence for all participants. We also applied a relatively prolonged (5-min) rest interval between bouts to minimize any accumulated ‘fatigue’. Most importantly, when we examine the data prior to each compression bout, , we reassuringly see that both heart rate and ratings of perceived exertion are similar across all exertional tasks (i.e., the data are fairly homogenous). As such, we are confident that fatigue played very little (if any role) in the data obtained. This could be because we recruited participants with a high level of experience who are required to be capable of providing prolonged (more than a single cycle) CPR and to remain consistent. This we hope may have further reduced the influence of fatigue due to the, hopefully, better chest compression technique within this group.*

*Notwithstanding the above, we agree that an alternative design may have been to undertake testing on separate days, however time constraints, participant commitment, work schedules and venue availability would have made this considerably more difficult. Similar recruitment constraints would have been met should a between-groups design been employed, where the required sample size in order to achieve a similar level of power within the study may have become prohibitively large.*

Suggestions:

1- Adding a descriptive table of the subject’s characteristics.

2- Adding a study figure that shows exactly what subjects went through and how many were in each group etc. Figure would make it easier to visualize study design to the reader. Procedure section.

*Thank you for these suggestions. We have included a supplementary table detailing the individual participant descriptives with the manuscript files. This has not been included in the main manuscript as it would duplicate existing information. Please see Supplementary Table 1.*

*As suggested, we have included a schematic figure of the experiment (Figure 1). We also hope that the removal of the Survey portion of the study from the manuscript also lends some clarity to the overall design.*

**Reviewer #3**

In my opinion Figure 1 is not strictly required and should be removed from the publication.

*Thank you for this suggestion, Figure 1 has been removed from the manuscript.*

Actual non-significant p values should be specified, rather than being provided as p>0.05

*Actual p-values have been added as requested.*

*Please see Lines: 151-154, 158-160, 184-186, 194-197.*

1) Was re-training attempted in the 5 females excluded from study. One would potentially hypothesize that their technique was inadequate and that re-training may have allowed participation in the study.
2) Consider adding the 5 females excluded as an "intention to treat" analysis. Did this make any difference to your reported study findings. (I note that this data has been collected as per the attachment in your offline data file provided

*Re-training for the purposes of the study was not attempted in the 5 excluded participants, although they were debriefed on their results following data collection such that they could address their technique. These participants were excluded on the basis that they were ‘unable to perform high-quality chest compressions under normal conditions’ (upon the floor). Specifically, they were unable to maintain a mean compression depth upon the Floor surface >40mm as was pre-defined prior to testing. As the primary question within our study was: “does safety matting influence chest compression quality”, we attempted to create the best possible conditions outside of a purely mechanically-based study to test the question. We therefore attempted to limit additional influences beyond that of the matting effect itself.*

*In our attempts to achieve this, we made substantial efforts to ensure compressions of a consistent quality among our participants, while trying to limit bias introduced by knowledge of the potential study outcomes. This included: i) recruiting participants trained to a high level, but also with significant levels of real-world experience, ii) prior to testing, briefly re-familiarising participants with the ‘feel’ of providing adequate chest compressions on a mannequin through the use of a brief mannequin-based CPR bout, with accompanying visual feedback, iii) allowing adequate rest periods between bouts, iv) blinding (in so far as was possible) to the individual surfaces tested using an opaque covering, and v) having pre-defined criteria for exclusion from the study. Even if scheduling a re-test had been easily feasible, it is likely that re-training these participants and allowing a further attempt, would have compromised our efforts to blind participants to the surface conditions.*

*In relation to the potential addition of these participants on an intention-to-treat basis (Comment 5), having now performed this analysis, our results and conclusions remain unchanged. These additional participants did not confer any alternative results within the data except in relation to a significant main effect for Heart Rate (p=0.024) between the LCBB and HC surfaces (126* ± *23 and 134* ± *24 bpm respectively). This does not influence any of our conclusions. Although we agree that these generally lighter and smaller-stature individuals represent an important group for future investigation, in the interests of basing our data on a group of participants delivering relatively homogenous, high-quality chest compressions, we have elected not to include the data attained by these excluded participants.*

*Line 280-282.*

Interesting that Table 2 describes statistically significant differences. However the 95th % CI overlap for those described as different. Additionally, the means of all parameters described are within 1 SD of the remaining parameters defined. The raw data and statistics should be carefully re-checked.

*As you correctly point out, Table 2 describes statistically significant differences between some surfaces (predominantly the high-compliance foam surface), accompanied by relatively tight standard deviations around the respective mean values. In order to ensure confidence in our results, we have returned to the original data exported from the mannequin into MS Excel and initially compared these to the mean data outputted within the mannequin’s proprietary software. In the first instance we can confirm that these are in agreement, within the limitations of the proprietary software only reporting to whole numbers, rather than three decimal places as was possible in the exported data.*

*Next, we ensured that the data transposed into SPSS from MS Excel was performed correctly, and after careful visual inspection, can confirm that this is the case.*

*Our analyses were then re-run within SPSS as either their respective repeated measures ANOVA with post-hoc Bonferroni correction (compression depth, rate and % ‘adequate’ compressions) or a 2-way repeated measures ANOVA (HR and RPE data) and the outputs re-examined.*

*Regarding the specific query relating to the overlap of the 95% CI for variables described as significantly different, the CI’s have been re-checked within MS Excel. For this, we used the =CONFIDENCE.T function which confirms the current means and CI’s as correct. We agree that when the CI’s of two means do not overlap, the difference is always significant. However, the converse (as in the case here) is not always true and even with overlapping CI’s, the difference between two means may still be statistically significant. We cite the enclosed paper by Austin and Hux (2002) which succinctly explains the issue at hand.*

*We are therefore happy that our data is correct, analyses have been performed appropriately and to the best of our knowledge are likely to represent accurate analyses and results.*

Reference:

Austin, Peter C., and Janet E. Hux. "A brief note on overlapping confidence intervals." *Journal of vascular surgery* (2002): 36(1); 194-195.
