# Supplementary material for: Sports safety matting diminishes cardiopulmonary resuscitation quality and increases rescuer perceived exertion
Source: PLoS One. 2021 Jul 22;16(7):e0254800. doi: 10.1371/journal.pone.0254800 (PMC8297772; doi:10.1371/journal.pone.0254800)
Supplement: Attachment — Submitted filename: Authors responses to review comments 29-6-21.docx [file pone.0254800.s005.docx]

**Responses to review comments 2:**

**Sports safety matting diminishes cardiopulmonary resuscitation quality and increases rescuer perceived exertion**

Thomas Kingston, Nicholas B. Tiller, Elle Partington, Mukhtar Ahmed, Gareth Jones, Mark I. Johnson, Nigel A. Callender.

We would like to thank the reviewers for providing comprehensive and informative feedback on the manuscript. Please find below their specific comments along with our annotated responses. We have tracked any revisions to the manuscript by including the relevant line numbers and highlighting changes in **blue** for ease of reference. Note some changes have been made to formatting at the request of the journal.

**Reviewer #1**

My previous concern about multiple objectives in a paper (including survey and research data together) has been resolved by the authors. However the opening line in the discussion section still mentions about "also describe current levels of preparedness for CPR delivery among 222 indoor climbing gymnasiums" Please change this to accurately reflect the current objective of the paper.

*Thank you for highlighting this errant line within the manuscript (Line 221). It has now been removed and the manuscript re-checked for any further outstanding issues. Thank you also for your time reviewing our work.*

**Reviewer #2**

No comments

*Thank you for your time and comments relating to our manuscript.*

**Reviewer #3**

Thank you for addressing my previous comments. I do not have further questions or comments.

*Thank you for your time and help in improving our manuscript.*
